# HIV research in Australia: linking basic research findings with clinical and public health outcomes

**DOI:** 10.1186/1742-4690-3-86

**Published:** 2006-12-01

**Authors:** Sharon R Lewin, John M Kaldor, David A Cooper

**Affiliations:** 1 Infectious Diseases Unit, Alfred Hospital, Melbourne, Australia; 2 Department of Medicine, Monash University, Melbourne, Australia; 3 National Centre for HIV Epidemiology and Clinical Research, Sydney, Australia

## Abstract

Despite a population of only 20 million and sustained low prevalence of HIV infection in Australia, Australian researchers have provided many substantial original findings to the fields of HIV pathogenesis, treatment and prevention. More recently, Australian clinicians and scientists have turned their attention to assisting other countries in developing effective responses, particularly within the Asia-Pacific region. It is therefore fitting that the 4th International AIDS Society (IAS) Conference on HIV Pathogenesis, Treatment and Prevention will be held in Sydney in July 2007. The meeting is expected to attract over 5000 participants and will have a dynamic and innovative programme within the three major themes of HIV basic science, clinical research and biomedical prevention.

## The HIV epidemic in Australia

Australia is generally viewed as a success story in the global HIV epidemic, with its national strategic response in place since the late 1980s credited with delivering extremely low infection rates and high levels of treatment access. Perhaps less well known is the extent to which Australia has been able to contribute to the world's knowledge about HIV infection, particularly in the areas of pathogenesis and clinical medicine. A unique combination of a geographically and demographically focussed epidemic, specialised medical units, a history of outstanding research in basic immunology and virology and dedicated national resources for health care and research has given rise to a scientific output that is disproportionate to the size of Australia's HIV epidemic.

Although Australia was one of the first countries in the Asia Pacific Region to report AIDS cases, and its epidemic curve rose sharply during the first half of the 1980s, it had begun to plateau by the early 1990s, and reached a peak even before the treatment revolution was under way. A rapid expansion of needle and syringe programs ensured that people who injected illicit drugs were largely protected from HIV infection, with prevalence consistently reported as being below 1% in this population. Nationally, the estimated prevalence is now among the lowest in the world, at around 0.1%

By far the most common cause of HIV transmission has been male to male sex, which has been associated with most of the cumulative 22,000 diagnoses estimated to have occurred in Australia since the first case in 1982. In the first decade of the epidemic, the proportion was even higher, and cases of HIV infection were highly concentrated in the large urban centres, where vibrant gay communities had developed in the late 1970s. In several cities, these communities were located near teaching hospitals with an established track record in virology and clinical infectious diseases, such as the Fairfield Infectious Diseases Hospital in Melbourne. At one stage it was estimated that 60% of the people with HIV infection in Australia lived in a five km radius of St Vincent's Hospital, Sydney.

In addition, Australia had a proud history of outstanding scientific research, particularly in the fields of virology and viral immunology that was well established prior to the arrival of HIV infection. Notable virologists such as Professor Frank Fenner and Professor Ian Gust, together with Nobel Prize winning immunologists Professor Macfarlane Burnet and Professor Peter Doherty had already influenced the generations of investigators who subsequently immersed themselves in HIV research. The national and State governments in Australia supported both the provision of services and the conduct of research, and it was this confluence of circumstances that fostered a dynamic culture of discovery across the spectrum of scientific disciplines involved in investigating HIV infection.

## Notable early discoveries

Some key early clinical research discoveries in Australia included the initial description of HIV seroconversion illness (an illness similar to the presentation of acute infectious mononucleosis or "glandular fever" which occurs within days to weeks following infection with HIV) [1]; the identification of HIV transmission by artificial insemination [2]; the transmission of HIV from mother to child not just during pregnancy but following delivery [3]; the predictive value of the CD4+ T-cell count in the development of particular opportunistic infections and malignancies in HIV-infected individuals [4]; and patient to patient transmission via inadequate sterilisation of surgical instruments used in an outpatient setting [5].

In 1992, a group of people in Sydney who had become infected with HIV following receipt of blood products from a single donor were identified as showing slow or no progression to AIDS [6]. The sequence of the virus that infected both the donor and recipients demonstrated multiple mutations in the U3 region of LTR overlapping the *nef *gene [Deacon, 1995]. The mutations in LTR-*nef *led to an attenuated virus that was less pathogenic than wild type virus. This observation strongly suggested that HIV Nef was a key viral determinant for disease progression in humans – a finding that had previously only been shown in monkeys infected with *nef*-deleted mutants of SIV. Subsequent work on the virology, immunology, neuropathology and clinical outcome of the Sydney blood bank cohort has led to over 30 publications, several patents and an ongoing productive research program on the study of non-progressive HIV infection.

## Novel clinical studies

Following the widespread introduction of highly active antiretroviral therapy (HAART) in 1996, came the first reports from Sydney of the clinical syndrome of lipodystrophy characterised by loss of fat in the periphery (face, buttocks, arms and legs), deposition of fat centrally (abdomen, breast and upper back) and associated metabolic disturbances including hypercholesterolemia, hypertriglyceridemia and insulin resistance [7]. Although, the mechanism of lipodystrophy was originally elusive, several Australian groups have identified a number of factors that contribute to the complex, multifactorial etiology of this syndrome. Some of these factors include the contribution of mitochondrial toxicity to fat wasting and the association of the lipodystrophy syndrome with specific classes of antiretroviral agents [8]. More recently, strategies to potentially reverse or treat these complications have been explored, although to date an effective treatment for this syndrome has proven difficult to develop [9].

Detailed characterisation of a relatively small cohort of HIV-infected individuals in Perth identified the association of hypersensitivity to the HIV reverse transcriptase inhibitor abacavir and carriage of the HLA-B5701 allele [10]. As a result of this significant finding, a simple screening test for this allele is now commonly performed prior to the use of abacavir. Abacavir is not prescribed to individuals who carry this allele and therefore screening can effectively prevent nearly all hypersensitivity reactions to this drug.

Perth researchers also published one of the first descriptions of potent immune restoration to opportunistic pathogens following the initiation of antiretrovirals – even just following the use of AZT, a relatively weak antiretroviral agent when used alone [11]. With the subsequent use of HAART, immune restoration disease (IRD) was then defined as a very important and common clinical syndrome. IRD occurs with a wide range of pathogens and the pathogenesis and genetic predisposition to development of IRD has since been well characterised [12].

## HIV basic research

Australia has also generated important basic science findings that have arisen from astute clinical observations and detailed study of clinical samples. The initial description of the association between clinical resistance to AZT and a mutation from leucine to tryptophan at position 210 in the reverse transcriptase was first reported from Melbourne in 1996 [13]. Australian researchers also made several key findings regarding the role of macrophages as a long term reservoir for HIV including in individuals receiving HAART [14]; the pathogenesis and prevalence of HIV-related dementia and other neurological complications [15-17] and our understanding of T-cell turnover in acute and established infection [18,19]. More recently, came the demonstration that HIV can bind to its co-receptor CCR5, with increasing efficiency at the later stages of infection, or when an individual progresses to AIDS [20]. This finding may potentially account for increasing pathogenicity of viral isolates in AIDS, without the use of alternate co-receptors such as CXCR4.

Investigators in Perth used sophisticated statistical algorithms to identify that polymorphisms in HIV were significantly associated with particular host HLA class I alleles and that absence of polymorphism was also HLA allele-specific [21]. Furthermore, at a population level, the degree of HLA-associated selection in viral sequence was predictive of HIV viral load. These results supported a fundamental role for HLA-restricted immune responses in driving and shaping HIV evolution in vivo and led to a significant change in thinking of how virus adaptation occurs.

Basic molecular investigations of HIV replication have also led to novel findings including a molecular basis for the role of Tat protein for fully efficient reverse transcription [22]; the intrinsic antiviral resistance of the double stranded (ds)RNA activated PKR system and its role in restricting HIV replication in astrocytes [23]; and critical steps in HIV RNA and reverse transcriptase dimerisation, packaging and virion assembly pathways [24,25].

Several groups in Australia have played a key role in our understanding of the interaction of HIV with different dendritic cell subsets [26,27]. Immature, mature and tissue-derived dendritic cells all express a range of receptors that can bind HIV and that are exclusive to this cell lineage. These receptors are all part of the c-type lectin family, are diverse in number and have differing affinity for binding to HIV [28], a finding that may have implications for the development of agents to inhibit the binding of HIV to these receptors and block sexual transmission.

## Innovative prevention strategies

Beyond the basic and clinical sciences, Australian researchers have also been active in epidemiological and social research related to HIV infection. The effectiveness of needle and syringe programmes in the prevention of HIV infection was demonstrated in a comprehensive analysis of international prevalence data [29]. Insights into sexual behaviour of gay men have crucially informed prevention strategies both nationally and internationally.

The development of effective biomedical prevention strategies is currently an active and growing area of research in Australia. Some novel approaches developed in Australia have included the production and evaluation of a "prime boost" preventative vaccine (DNA priming with fowlpox boost) which was shown to be strongly immunogenic in primates [30]. Although less immunogenic in humans, the administration and synthesis of these constructs is currently being optimised and this prime boost vaccine will be evaluated in phase II human studies in Thailand in the next year. Another novel strategy recently shown to induce HIV-specific T-cell responses in primates is the infusion of small overlapping peptides that match the HIV consensus sequence together with autologous whole blood [31]. This strategy could potentially be used in humans as a therapeutic or prophylactic vaccine. Australian based biotechnology companies have also been active in the development of new agents with the potential for use as vaginal microbicides.

Despite some indications of an upturn in infection rates over the past few years, the epidemic in this country has been largely stable, and Australian clinicians and scientists have increasingly turned their attention to assisting other countries in developing effective responses. Within the Asia-Pacific region, Australia has played a key role in the development of clinical investigation and disease surveillance, though research collaborations in Thailand and Cambodia, and donor funded bilateral programs in extremely resource-poor countries such as Myanmar and more recently Papua New Guinea. The Burnet Institute based in Melbourne has played a major role in the advocacy, introduction and maintenance of needle syringe programs throughout the region.

## IAS 2007 in Sydney

Given Australia's significant contribution to our understanding of HIV pathogenesis and prevention and its emerging leadership role within the Asia-Pacific region, it is fitting that the 4th International AIDS Society (IAS) Conference on HIV Pathogenesis, Treatment and Prevention will be held in Sydney in July 2007 [32]. The IAS Conference on HIV Pathogenesis, Treatment and Prevention is one of the leading international conferences for researchers in all scientific fields related to HIV – basic science, virology, immunology, epidemiology, clinical management and pharmacology.

The local host for the conference is the Australasian Society for HIV Medicine (ASHM). ASHM is Australasia's peak body representing the HIV medical and research communities. The society incorporated in 1990 and was one of the first National HIV/AIDS societies in the world, and an early member of the International AIDS Society. It has successfully run an annual scientific conference on HIV/AIDS since 1989. This year, over 1000 delegates attended the 19^th ^ASHM annual conference in Melbourne.

This is the first time an HIV-related conference of this magnitude will be held in Australia. Over 5000 delegates from over 150 countries are expected to attend. We are planning an exciting and innovative program that will highlight cutting edge research in each of the three major themes of basic science, clinical research and biomedical prevention. We encourage established researchers, post-doctoral fellows and graduate students interested in HIV and AIDS to attend IAS 2007 and enjoy the science, Sydney and Australia.

## Competing interests

Financial: nil

Non-financial: DAC is Local Chair and SRL and JMK Local Deputy Chairs for the 4^th ^International AIDS Society Conference on HIV Pathogenesis, Treatment and Prevention. SRL is President of the Australasian Society of HIV Medicine.

## Authors' contributions

All authors contributed equally to the manuscript.
